# Mangosteen Pericarp and Its Bioactive Xanthones: Potential Therapeutic Value in Alzheimer’s Disease, Parkinson’s Disease, and Depression with Pharmacokinetic and Safety Profiles

**DOI:** 10.3390/ijms21176211

**Published:** 2020-08-27

**Authors:** Ha Thi Thu Do, Jungsook Cho

**Affiliations:** College of Pharmacy, Dongguk University-Seoul, Dongguk-ro 32, Ilsandong-gu, Goyang, Gyeonggi 10326, Korea; doha201191@gmail.com

**Keywords:** mangosteen (*Garcinia mangostana* L.) pericarp, neurodegenerative diseases, Alzheimer’s disease, Parkinson’s disease, depression, α-mangostin, γ-mangostin, pharmacokinetics, safety

## Abstract

Alzheimer’s disease (AD), Parkinson’s disease (PD), and depression are growing burdens for society globally, partly due to a lack of effective treatments. Mangosteen (*Garcinia mangostana* L.,) pericarp (MP) and its xanthones may provide therapeutic advantages for these disorders. In this review, we discuss potential therapeutic value of MP-derived agents in AD, PD, and depression with their pharmacokinetic and safety profiles. MP-derived agents have shown multifunctional effects including neuroprotective, antioxidant, and anti-neuroinflammatory actions. In addition, they target specific disease pathologies, such as amyloid beta production and deposition as well as cholinergic dysfunction in AD; α-synuclein aggregation in PD; and modulation of monoamine disturbance in depression. Particularly, the xanthone derivatives, including α-mangostin and γ-mangostin, exhibit potent pharmacological actions. However, low oral bioavailability and poor brain penetration may limit their therapeutic applications. These challenges can be overcome in part by administering as a form of MP extract (MPE) or using specific carrier systems. MPE and α-mangostin are generally safe and well-tolerated in animals. Furthermore, mangosteen-based products are safe for humans. Therefore, MPE and its bioactive xanthones are promising candidates for the treatment of AD, PD, and depression. Further studies including clinical trials are essential to decipher their efficacy, and pharmacokinetic and safety profiles in these disorders.

## 1. Introduction

Neurodegeneration is described as the progressive loss of neurons in the central nervous system (CNS) with consequent clinical deficiencies. Major neurodegenerative diseases, such as Alzheimer’s disease (AD) and Parkinson’s disease (PD) are medical and economical burdens for patients, caregivers, and society worldwide [1]. AD is the most common cause of dementia, which is characterized by progressive memory loss, language problems, and defective cognitive abilities [2]. There are consistent age-dependent increases in the incidence of AD, from approximately 0.5% per year among individuals aged 65–70 to approximately 6–8% among individuals over 85 years old [3]. Notably, AD is reported to be the fifth leading cause of death for individuals over 65 years of age in the United States (U.S.) [2]. In addition, PD is the most common movement disorder and second most prevalent neurodegenerative disease after AD. PD is typically identified by motor symptoms, such as resting tremors, muscle rigidity, bradykinesia, and postural instability; and non-motor symptoms, including depression, sleep disturbances, and cognitive decline [4]. By 2016, PD prevalence had increased to 6.1 million people worldwide from 2.5 million in 1990 [5]. It affects approximately 1–2% of individuals over 65 years of age [4].

Depression, a mood disorder that can interfere with daily functioning, is another significant cause of global burden [6]. It is described as persistent feelings of sadness, a loss of pleasure or interest, tiredness, disturbed appetite and sleep, poor concentration, feelings of guilt and low self-worth, and suicidal ideation [7]. In 2017, depression was the leading cause of disability worldwide, affecting more than 264 million people [8]. In addition to the high prevalence, the lack of effective treatments for AD, PD and depression significantly augment the disease burden. Therefore, new remedies or conjunctive treatments for these disorders are unmet needs for future research. Growing evidence has indicated that natural products may provide therapeutic advantages to these brain diseases [9,10,11]. In the present review, we will focus on the potential therapeutic value of various agents derived from mangosteen.

**Figure 1:**
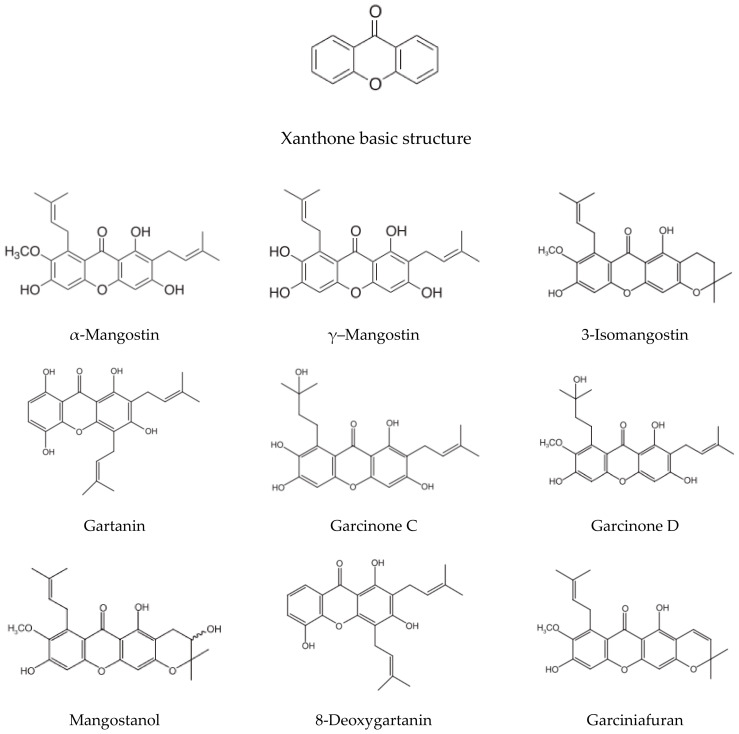
Chemical structures of basic xanthone and representative bioactive xanthones in mangosteen pericarp.

Mangosteen, *Garcinia mangostana* L., is a tree cultivated mostly in Southeast Asian countries. Mangosteen fruit, known as ‘‘the queen of fruits”, is edible, with a sweet and tangy taste. Some mangosteen-based products, such as juice and tablets, are among the best-selling food supplements in the U.S. [12]. Additionally, mangosteen is well-recognized for its medicinal benefits. Mangosteen pericarp (MP) has long been used as a traditional medicine to treat infection, wounds, inflammation, and diarrhea [13]. Phytochemical studies have explored medicinal properties using xanthone derivatives—the major bioactive secondary metabolites derived from MP. Xanthones are polyphenolic compounds, which are characterized with a distinctive skeleton of a xanthene-9-one that are distinguished by the tricyclic aromatic ring system (Figure 1) [14]. At least 68 distinct xanthones, which exist as oxygenated or prenylated forms, have been identified in different portions of the plant, with more than 50 xanthones being present in MP at higher concentrations than in other parts of the fruit [14]. The most investigated xanthones in MP are α-mangostin (α-MG) and γ-mangostin (γ-MG). Other xanthones found in MP include β-mangostin, gartanin, 3-isomangostin, garcinones A, B, C, D, and E, 8-deoxygartanin, mangostanol, isomangostin, and garciniafuran [14,15]. The chemical structures of representative bioactive xanthones are shown in Figure 1. MP and its bioactive xanthones have been reported to exhibit numerous biological activities, including antiparasitic, antitumor, antioxidant, anti-inflammatory, and neuroprotective effects in various experimental studies [16].

MP and xanthones derived from MP have recently attracted researchers’ attention for their pharmacological actions in neurons. Firstly, MP-derived xanthones such as garcinone D or gartanin exerted neuroprotective activities and mitigated oxidative injury, possibly via Nrf-2/HO-1 and STAT3/Cyclin D1 [17], or AMPK/SIRT1/PGC-1α signaling pathways [18], respectively. In addition, the xanthone derivatives exhibited anti-inflammatory effects in the brain [19,20]. Besides, the xanthones alleviated lead-induced neurotoxicity in mice via suppression of oxidative damage and reversion of acetylcholinesterase (AChE) activity, resulting in the restoration of learning deficits and memory loss [21]. Several studies have reported the neuroprotective and antioxidant properties of α-MG via the amelioration of mitochondrial toxin 3-nitropropionic acid or iodoacetate-induced reactive oxygen species (ROS) production in primary culture of cerebellar granule cells [22,23]. Intriguingly, the polar fraction of ethanol MP extract (MPE) augmented antioxidant capability and attenuated oxidative damage to proteins in the blood cells of healthy adults, demonstrating its antioxidant effects in humans [24].

With the neuroprotective, antioxidant, anti-neuroinflammatory activities, MP-derived agents such as MPE and its bioactive xanthones may exert beneficial actions in many brain diseases. Wang et al. provided a comprehensive review on the pharmacological properties of mangostins and their derivatives, which promised their potential utilities in the treatment of certain diseases including AD [25]. In addition, Ashton et al. extensively reviewed the potential of MP as an adjunctive therapy for serious mental illnesses such as bipolar disorder and schizophrenia [26]. In the present study, we will discuss the potential therapeutic value of MP and its bioactive xanthones, particularly focusing on the treatment of AD, PD and depression. We first provide a concise overview of the major pathologies and therapeutic strategies of AD, PD, and depression, and then discuss pharmacological effects of MP-derived agents in these diseases along with their pharmacokinetic and safety profiles.

## 2. Major Pathologies and Therapeutic Strategies of AD, PD, and Depression

From a neuropathological point of view, AD, PD, and depression involve specific pathologies associated with each disorder, but also share several common ones. In this section, we briefly describe specific and common pathologies associated with AD, PD, and depression. In addition, we discuss the current treatments of these diseases and propose potential therapeutic strategies for future research.

AD is a slowly progressive neurodegenerative disease that begins many years before symptoms emerge. The most widely accepted hallmarks of the clinical features of AD are: (1) the formation and deposition of extracellular amyloid beta (Aβ) plaques and (2) the accumulation of intracellular hyperphosphorylated tau proteins, known as neurofibrillary tangles, in the brain [2]. Aβ is a short peptide, with its most common alloforms being Aβ_1-40_ and Aβ_1-42_, derived from the cleavage of an amyloid precursor protein by β- and γ-secretases [27,28,29]. As a consequence, Aβ peptides spontaneously form soluble oligomers, coalesce into insoluble beta-sheet conformation, and are extracellularly deposited in diffuse senile plaques [30]. This process interferes with neuron-to-neuron communication at synapses, and leads to neuronal cell death [2]. Tau protein is involved in the assembly and stabilization of microtubules in neurons [31]; however, the hyperphosphorylation of tau causes the loss of microtubule stabilization, which leads to the abnormal shape and functionality of neurons, and eventually, neuronal cell death [32,33]. In addition, Aβ peptides and hyperphosphorylated tau destabilize calcium homeostasis and increase the vulnerability of neurons to excitotoxicity, mainly via interactions with N-methyl-D-aspartate (NMDA) receptors [34,35]. These processes eventually induce the impairment in cognitive and memory function seen in AD patients [32,36]. Moreover, the intellectual deficiency in AD patients has been linked to a loss of cholinergic function in the cortex and hippocampus, which also contributes to AD pathology [37]. To date, the approved drugs for AD treatment are cholinesterase inhibitors and a NMDA receptor antagonist. Numerous clinical trials were performed so far to improve the cognitive and behavioral symptoms of AD [38]. In particular, disease-modifying therapies targeting Aβ or tau protein, neuroprotective strategies, and immunotherapies have been intensely attempted. Unfortunately, however, due to extremely low success rates, there has been no new approved drug for the treatment of AD since 2003 [38].

PD is characterized by a preferential loss of dopaminergic (DAergic) neurons within the substantia nigra pars compacta (SNpc) and the deposition of intracellular inclusions, known as Lewy bodies [39,40]. Lewy bodies are predominantly formed by misfolding and aggregation of the presynaptic protein, *α*-synuclein (*α*-syn), in surviving neurons [39,40]. Presynaptic compensation in the nigrostriatal dopamine (DA) pathway may account for PD progression. Typically, parkinsonian motor symptoms become apparent when more than 30% of nigral DA neurons or 50% of striatal DA contents are lost [41,42]. Current PD treatment primarily consists of DA replacement therapies, such as levodopa, DA agonists, and inhibitors of catechol-O-methyl transferase and monoamine oxidase (MAO) B. Additionally, anticholinergics and amantadine are used to ameliorate motor symptoms, while antipsychotics and antidepressants are prescribed to treat non-motor symptoms of PD [43,44].

Monoamine dysregulation is recognized as a primary pathology of depression. DA and serotonin (also known as 5-HT) are dysregulated in depression. DAergic and serotonergic functional loss in the striatum and hippocampus, respectively, is associated with disturbances in mood regulation, which is linked to depressive disorder [45,46,47]. Moreover, downregulation of norepinephrine (NE) [48] and disturbances in γ-aminobutyric acid (GABA) and glutamate are also recognized in this disease [49]. Since monoamine imbalance is the primary pathology of depression, it has been the target of pharmacological treatments. Modulation of DA, serotonin, and NE imbalance is the major mechanisms of action by which antidepressant drugs exert their actions. The major antidepressants include selective serotonin reuptake inhibitors, serotonin-NE reuptake inhibitors, NE-DA reuptake inhibitors, 5-HT_2_ receptor antagonists, MAO inhibitors, and tricyclic antidepressants (TCAs) [50].

In addition to the specific pathologies associated with AD, PD, or depression as discussed above, these disorders share some pathologies in common. The common pathologies associated with AD, PD, and depression include neuroinflammation, oxidative stress, mitochondrial dysfunction, and reduction of neurotrophic factors such as brain-derived neurotrophic factor (BDNF). Neuroinflammation is the process of innate or adaptive immune activation occurring in the CNS in response to the perturbed homeostasis [51]. Overactivation of the immune system during neuroinflammatory processes may lead to neuronal damage in AD and PD [52,53,54]. Likewise, there is growing evidence supporting the involvement of inflammation in depression [55,56,57]. One of the potential underlying mechanisms is the suppression of serotonin synthesis from tryptophan by pro-inflammatory cytokines through activation of indoleamine 2,3-dioxygenase, a rate-limiting enzyme involved in tryptophan metabolism [58]. Oxidative stress is also a common feature of AD, PD and depression. Free radicals produced by oxidative stress affect the structure and function of neuronal cells, contributing to the development of AD and PD [59,60]. Elevated levels of ROS and nitrogen species are associated with increased damage of intracellular biomolecules such as DNA in the depressed brain [61]. Another study suggests that depression correlates with oxidative alterations of nucleotides and polymorphisms of genes associated with metabolism of ROS [62]. These findings strongly support critical roles of oxidative stress in the pathologies of AD, PD, and depression. Due to its direct relationship with oxidative stress, mitochondrial dysfunction also plays a key role in the pathogenesis of AD [63], PD [64], and depression [61,65]. Meanwhile, neurotrophic factors such as glial cell-derived neurotrophic factor, nerve growth factor, and especially, BDNF are crucial for survival, maintenance, and regeneration of specific neurons in the adult brain [66]. Reductions of these neurotrophic factors are commonly recognized in AD and PD [66,67,68] as well as in depression [69]. Therefore, agents targeting these common features may possess promising therapeutic values for these diseases.

Unfortunately, however, the current therapies for AD, PD and depression are not fully adequate for growing treatment needs. The drugs clinically used for the treatment of AD or PD show limited efficacy with only slowing the disease progress or just alleviating symptoms [38,70,71]. Similarly, current therapies for depression exhibit a number of limitations such as delayed onset of action, insufficient efficacy, and unwanted side effects [72]. Therefore, novel effective agents are urgently required to enter the therapeutic pipeline. New agents with neuroprotective, anti-inflammatory, and antioxidant properties or targeting AD-, PD-, or depression-related specific pathologies would be promising for curative outcomes in these diseases. In the following sections, we assess natural MP-derived agents as promising candidates to intervene in AD, PD and depression.

## 3. Pharmacological Effects of MP-Derived Agents in AD Models

Agents derived from MP have been shown to exhibit neuroprotective, anti-inflammatory, and antioxidant effects, supporting their efficacy as potential treatments or co-adjuvant therapies for brain disorders including AD.

### 3.1. Pharmacological Effects of MPE and MP Diet in AD Models

Various preparations of MPEs and MP diet were found to inhibit neurotoxicity and oxidative stress induced by Aβ, NMDA, H_2_O_2_ or other stimuli in in vitro and in vivo studies. An in vitro study revealed that pretreatment with the water-soluble partition of methanol MPE successfully prevented Aβ_1-42_-induced cytotoxicity, and reduced ROS levels and caspase-3 activity in SK-N-SH neuroblastoma cells. Interestingly, the MPE diminished Aβ_1-42_-induced changes of several proteins including FK506-binding protein 4 and HLA-B associated transcript 1, suggesting that these proteins might be responsible for the neuroprotective effects of MPE [73]. Moreover, the water extract of MP and the butanol fraction of methanol MPE significantly attenuated NMDA- and Aβ_25-35_-induced neuronal cell damage in primary cultured rat cortical neurons, possibly via inhibition of ROS generation and apoptotic events. In parallel, the antioxidant properties of these extracts and their inhibitory effects on β-secretase activity were also reported based on the in vitro studies [74,75]. In another study, the water and 50% ethanol MPE exhibited potent antioxidant effects with high free-radical scavenging and neuroprotective actions in H_2_O_2_-treated NG108-15 neuroblastoma cells [76]. Similarly, the water-soluble partition of ethanol MPE partially antagonized the effects of H_2_O_2_ and polychlorinated biphenyls on cell viability, ROS production, and caspase-3 activity in SK-N-SH cells [77]. The MPE was demonstrated to improve scopolamine-induced memory dysfunction in mice, as assessed by Morris water maze (MWM) and passive avoidance tests. Moreover, the increased ROS level and caspase-3 activity in the brain of scopolamine-treated mice were inhibited by the MPE treatment [77]. Besides, MP diet exerted neuroprotective and antioxidant effects in triple transgenic AD (3×Tg-AD) mice, and reduced Aβ deposition in the hippocampus, which might further attenuate the deficit in spatial memory retrieval in the MWM test [78]. Furthermore, 50% ethanol MPE significantly enhanced the antioxidant parameters, such as superoxide dismutase, glutathione peroxidase, glutathione, and catalase in the brain tissues of streptozotocin-treated male Swiss albino (SA) mice [79].

Several studies have demonstrated inhibition of AChE activities by MPE. Notably, the water-soluble partition of ethanol MPE reduced AChE activity of SK-N-SH cells to ~ 60% of the control activity [77]. The suppression of AChE by the water MPE was confirmed in another study [75]. Furthermore, pretreatment of male SA mice with 50% ethanol MPE prior to streptozotocin injection induced a dose-dependent reduction in AChE activities in the brain, and increased habituation memory and cognitive function as measured by open field test (OFT) and Y-maze test [79].

The effects of MP on the regulation of BDNF expression, tau phosphorylation, and neuroinflammation have been reported [78]. MP significantly decreased the cell death and increased BDNF expression in an organotypic hippocampal slice culture. In addition, 8-month dietary supplementation with MP significantly attenuated the cognitive impairment in aged C57BL/6J (B6) mice, primarily associated with anti-inflammation, increased BDNF level and decreased phosphorylation of tau. The MP diet in 3×Tg-AD mice also exerted anti-inflammatory effects and reduction of phosphorylated tau in the hippocampus, which might further be reflected in the improvement of spatial memory retrieval [78]. 

These data highlight that different preparations of MPE and MP diet have multiple pharmacological effects targeting specific and common AD pathologies including: prevention of Aβ production, deposition, and toxicity; inhibition of tau hyperphosphorylation, NMDA excitotoxicity, and AChE activity; and antioxidant and anti-inflammatory activities. Furthermore, improved cognitive and memory functions by MPEs and MP diet have been established in different AD animal models. Therefore, these agents may be beneficial for the prevention or treatment of AD. The pharmacological effects of MP-derived agents in various in vitro and in vivo AD models are summarized in Table 1.

### 3.2. Pharmacological Effects of Xanthones Isolated from MP in AD Models

Bioactive compounds derived from MP were shown to mitigate the neurotoxicity induced by glutamate or Aβ, and diminish Aβ production and aggregation. Xanthones such as α-MG, γ-MG, gartanin, and garcinone C exhibited neuroprotective effects against glutamate-caused HT22 hippocampal neuronal cell death, partly by up-regulating HO-1 protein level. Additionally, these xanthones suppressed self-induced Aβ aggregation and β-secretase activity [80]. In particular, α-MG was reported to diminish Aβ_1-40_ and Aβ_1-42_ production in primary cultured rat cortical neurons through inhibition of β- and γ-secretase activities in the amyloidogenic pathway [81]. Consistently, α-MG concentration-dependently attenuated the neurotoxicity induced by Aβ_1-40_ or Aβ_1-42_ oligomers in the cultured neurons. It was speculated that α-MG may disturb the pre-formed Aβ fibrils by stabilizing its α-helical conformation, and thereby, block the fibril formation [82].

Additionally, the xanthones were found to contribute to the antioxidant effects of MP in the brain. α-MG, γ-MG, gartanin, and garcinone C showed promising antioxidant properties with scavenging activities of 2,2-diphenyl-1-picrylhydrazyl (DPPH) free radicals. Intriguingly, however, only γ-MG, not α-MG, predominantly protected cultured cortical neurons against either H_2_O_2_- or xanthine/xanthine oxidase-induced oxidative neuronal death, and mitigated ROS generation [83]. γ-MG significantly abolished H_2_O_2_-induced DNA fragmentation and caspases-3 and -9 activation, indicating its anti-apoptotic action. In agreement with these findings, only γ-MG exhibited effective suppression of lipid peroxidation and DPPH radical formation in cell-free bioassays. In contrast, *β*-secretase activity was inhibited by both α-MG and γ-MG [83]. Remarkably, oral administration of γ-MG significantly reversed scopolamine-induced memory deficits in mice. These in vitro and in vivo findings suggest that γ-MG may be the preferable candidate over α-MG in combatting oxidative stress-associated neurodegenerative diseases including AD [83].

Prenylated xanthones derived from MP, including α-MG, γ-MG, mangostanol, 3-isomangostin, and garcinone C, showed inhibition of AChE activities in vitro [84]. Interestingly, molecular docking studies revealed that α-MG, γ-MG and garcinone C may interact differently with the key regulatory regions of AChE, mainly through hydrophobic interactions and hydrogen bonding. These promising results should be further investigated in animal studies [84].

As previously discussed, neuroinflammation is considered as one of the common pathologies associated with AD, PD [52,53,54] and depression [55,56,57,58]. It was demonstrated that oral gavage of α-MG significantly attenuated the lipopolysaccharide (LPS)-induced brain inflammation in B6 mice [20]. α-MG suppressed the brain levels of LPS-induced pro-inflammatory cytokine interleukin-6 (IL-6), cyclooxygenase-2, and 18 kDa translocator protein, a sensitive biomarker of brain inflammation [85]. The anti-inflammatory effects of α-MG may contribute to beneficial impacts on not only AD, but also PD and depression.

Collectively, the above findings demonstrated the multiple pharmacological effects of the MP-derived xanthones, especially α-MG and γ-MG, which could contribute to the treatment of AD (Table 1). Specifically, the compounds attenuated Aβ production, dissociated the Aβ aggregation, and thereby, inhibited Aβ-induced neurotoxicity. Furthermore, the xanthones were shown to suppress oxidative stress, as well as alleviate AChE activities. These findings illustrated the utility of natural xanthones as novel neuroprotective candidates through the intervention of multiple pathological processes of AD.

## 4. Pharmacological Effects of MP-Derived Agents in PD Models

Since AD and PD share common pathological features, the MP-derived products can also be potential candidates for PD treatment. Unfortunately, no studies have reported the effects of MPE on PD. However, there are several studies illustrating the pharmacological effects of α-MG and γ-MG in PD models. In this section, we discuss the potential value of these xanthones in PD treatment.

α-MG has been shown to exert neuroprotective and antioxidant functions in in vitro models of PD. Evidence showed that α-MG alleviated 1-methyl-4-phenylpyridinium-induced apoptosis in SH-SY5Y neuroblastoma cells, which may be associated with inhibition of ROS formation, modulation of the balance between pro- and anti-apoptotic proteins, and suppression of caspase-3 activation [86]. Another study showed that α-MG significantly ameliorated rotenone-induced cytotoxicity in SH-SY5Y cells in a concentration-dependent manner [87]. α-MG treatment also reversed rotenone-induced overproduction of ROS, activation of caspases-3 and -8, and mitochondrial dysfunction [87]. Notably, α-MG protected DAergic neurons from α-syn-induced neurotoxicity in primary rat mesencephalic neuron-glia co-cultures. Inhibition of ROS production by α-MG was also observed in α-syn-stimulated primary rat microglia [88].

The effects of α-MG on PD were further illustrated in vivo. Intraperitoneal (i.p.) injection of α-MG to adult male Sprague Dawley (SD) rats significantly restored rotenone-reduced locomotor activity and neuromuscular weakness, as assessed by rotarod and grip strength tests, respectively. Furthermore, treatment with α-MG demonstrated antioxidant effects in the striatum of the rats by diminishing rotenone-induced oxidative damage, such as increased malondialdehydes, nitrite concentrations, and decreased glutathione levels [89]. Importantly, α-MG inhibited α-syn aggregation and rescued tyrosine hydroxylase (TH), a rate-limiting enzyme involved in the synthesis of DA. Specifically, α-MG alleviated rotenone-induced aggregation of α-syn and loss of TH in SH-SY5Y cells [87]. Furthermore, α-MG also significantly reduced phosphorylation of α-syn, and thereby protecting from TH^+^-DAergic neuronal loss in the SNpc of the rat model of PD [89].

Considering the hydroxyl groups in the structure (Figure 1), γ-MG is also expected to possess free radical scavenging effects. Unfortunately, only a few studies have reported neuroprotective effects of γ-MG in PD models. γ-MG pretreatment was found to attenuate 6-hydroxyDA-induced neuronal cell death. The protective effect of γ-MG was associated with its antioxidant potential and modulation of apoptotic signaling [90]. These results illustrate the immense potential of γ-MG for preventing oxidative stress-induced neurodegeneration in PD.

It is well-known that microglial activation can initiate a cascade of events associated with neuroinflammation, which leads to progressive neurodegeneration of nigral DAergic neurons. Therefore, inhibitions of microglial activation and neuroinflammation are also attractive strategies for PD treatment [91]. As described earlier, orally administered α-MG suppressed the LPS-induced inflammation in the brain of B6 mice [20]. In addition, α-MG abated the increased levels of pro-inflammatory cytokines, including tumor necrosis factor-α, IL-1β, IL-6, and nitric oxide, in α-syn-stimulated primary rat microglia, possibly through the inhibition of nuclear factor kappa B and NADPH oxidase [88]. Although detailed mechanisms remain to be further defined, these results suggest that α-MG can be a promising therapy for PD.

Altogether, the above findings indicate that α-MG and γ-MG possess neuroprotective, antioxidant, and anti-inflammatory activities. Additionally, these xanthones were shown to arrest α-syn aggregation, TH loss, and mitochondrial dysfunction, illustrating their therapeutic roles in preventing PD progression. The pharmacological effects of these xanthones in PD models are summarized in Table 2. Further mechanistic studies of α-MG, γ-MG, and other bioactive compounds or extracts from MP are required on both preclinical and clinical levels to probe their potential value in the management of PD.

## 5. Pharmacological Effects of MP-Derived Agents in Depression Models

The tricyclic structure of xanthones in MP (Figure 1) may link to antidepressive functions as seen in TCAs. As described above, MP-derived agents exhibit redox-modulatory actions [18,22,23], anti-inflammatory effects [19,20], and probable reversion of mitochondrial dysfunction [87]. These effects may contribute to beneficial actions to surmount common pathologies of AD and PD as well as depression. However, limited evidence is available to support antidepressant effects of MP-derived agents.

Recent preclinical evidence has illustrated the antidepressant-like and pro-cognitive effects of MPE in Flinders Sensitive Line (FSL) rats, a genetic model of depression [92]. Acute administration of ethyl acetate MPE demonstrated antidepressant-like effects in OFT and forced swim test (FST), with the comparable effects to a reference antidepressant, imipramine (IMI, a TCA). Similarly, chronic treatment with this MPE displayed significant antidepressant- and pro-cognitive effects in FST and novel object recognition test, respectively. Again, the antidepressant effect of the MPE was comparable to that of IMI. Interestingly, however, the neurotransmitters associated with antidepressant effects of chronically treated MPE and IMI appeared to be different. A more prominent serotonergic action for MPE was observed as opposed to a noradrenergic action for IMI treatment. IMI significantly altered cortico-hippocampal NE levels, possibly by chronic blocking the NE transporter, which was consistent with a previous study [93]. In contrast, chronic MPE-treated FSL rats showed elevated levels of 5-hydroxyindoleacetic acid, a main metabolite of 5-HT, in both the frontal cortex and hippocampus, indicating serotonergic action. Numerous animal studies have shown the associations of oxidative stress with cognitive impairment [94,95] and depressive-like behaviors [96]. Indeed, reversion of hippocampal lipid peroxidation was observed with IMI or MPE treatment. These findings indicate that modulation of dysregulated brain monoamines and suppression of oxidative stress are possibly involved in the antidepressant activity of MPE [92]. Another study revealed that α-MG markedly exhibited antidepressant-like activity, which was reversed by pretreatment with haloperidol (a D_2_ receptor antagonist), *p*-chlorophenylalanine (an inhibitor of 5-HT synthesis), and bicuculline (a competitive GABA antagonist). Furthermore, α-MG effectively accentuated brain DA, 5-HT and GABA levels in mice, indicating that these neurotransmitter systems may mediate antidepressant-like effect of α-MG [97]. As mentioned previously, orally administered α-MG exhibited anti-inflammatory effects in B6 mice, which may also contribute to beneficial impacts on depression [20]. In addition, both ethyl acetate MPE and α-MG revealed significant antidepressant-like properties in a rat model of schizophrenia, suggesting that these agents may specifically address the depressive symptoms of schizophrenia [98].

Although there are no clinical data directly focusing on the effects of MP agents in the depressive patients, several studies have evaluated their impacts on mental health outcomes in humans. In a small randomized controlled trial (RCT), mangosteen-based supplementation containing 305 mg of α-MG and 278 mg of hydroxycitric acid was orally administered to healthy adults prior to cycle ergometer exercise and the parameters directly reflecting the condition of physical fatigue were measured [99]. All parameters measured showed no differences between the supplementation group and placebo group, except the Profile of Mood States (POMS) scores. The single dose of mangosteen supplementation showed a positive impact on the scores of POMS, a psychological rating scale used to assess transient, distinct mood states [99]. 

Intriguingly, α-MG was found to reduce body weight (b.w.) gain in high-fat diet-induced obese mice [100]. Moreover, there are several clinical results exhibiting the efficacy of mangosteen-based products in obese individuals. Among them, a RCT assessed the efficacy and tolerability of Meratrim, an herbal formulation consisting of extracts from *Sphaeranthus indicus* flower heads and MP mixed together in a ratio of 3:1, in controlling b.w. in healthy overweight subjects [101]. In this study, while the reduction of b.w. from the baseline to the end of 16 weeks-treatment period was evaluated as a primary endpoint, total mood disturbance (TMD) scores were also measured as a secondary endpoint using POMS-short form. Notably, a statistically significant decrease in the mean TMD score was reported in subjects receiving Meratrim over placebo [101]. In addition, a pilot study of double-blind placebo-controlled trial was conducted using encapsulated MP powder in 80 patients with schizophrenia or schizoaffective disorder [102]. As the primary outcomes, significant reduction of symptom domains associated with schizophrenia was observed in the MP group. Furthermore, secondary outcomes, assessed by Montgomery Åsberg Depression Rating Scale to measure the severity of depressive episodes at 180-day endpoint, were also significantly different between the two groups. The findings from these randomized trials demonstrate that MP has beneficial impact on depression. However, this study possesses several limitations; small sample size and participants with mild depression at baseline [26]. Nevertheless, this is the only clinical study which directly evaluates the potential therapeutic value of MP in the treatment of a serious mental illness accompanied by depression. These positive results may initiate further research of MP for the treatments of depression and other psychiatric disorders with depression. In fact, one RCT (Trial registry ID: ACTRN12616000028404) is currently in progress investigating the efficacy of adjunctive MP for bipolar depression [103].

Taken together, both MPE and α-MG showed the antidepressant activities via modulation of dysregulated monoamines and oxidative stress in the brain, suggesting novel potential therapies for depression. The pharmacological effects of the MP-derived agents in depression models are summarized in Table 3. However, since our knowledge regarding the underlying mechanisms associated with the antidepressant effects of MPE, α-MG, or other xanthones in MP remains poor, further investigation is required.

## 6. Pharmacokinetic (PK) Profiles of MP-Derived Agents

We discussed in vitro and in vivo pharmacological effects of MP-derived agents in the experimental models of AD, PD, or depression in the previous sections. The MPE and several xanthones including α-MG are described to be promising candidates for the treatment of AD, PD, and depression. In the following section, we discuss the current evidence of the PK profiles of MP-derived agents, including commercially available MP-based products, in the preclinical and clinical conditions and propose solutions to improve their PK profiles.

### 6.1. In Vitro and In Vivo PK Profiles of MP-Derived Agents

PK studies for herbal agents containing multicomponent mixtures with numerous active phytochemicals are generally extraordinarily complex [104], which may explain the lack of PK data for MP-derived agents. Intravenous (i.v.) injection of α-MG exhibited a biphasic deposition with a fast distribution phase and a slow terminal elimination phase, suggesting high tissue binding in rats [104]. The bioavailability after oral administration of α-MG was very low, probably due to a poor gastrointestinal absorption or an extensive metabolism in the small intestine and liver [104,105]. The distribution of α-MG was relatively high in the intestine, liver, kidney, and lung, but not in the brain. Interestingly, however, it was reported that some xanthones such as γ-MG, gartanin, and garcinone C were able to penetrate the blood-brain barrier (BBB) in vitro [80]. It was proposed that α-MG underwent phase II, and possibly, phase I metabolism [105,106]. Although α-MG was metabolized primarily by CYP1A2 in vitro [107], an in vivo study indicated that phase I metabolism played a minimal role in the overall metabolism of α-MG. Phase II metabolism appeared to be the primary route, which was consistent with previous reports [105]. To date, only one study has measured the absolute bioavailability of γ-MG; this study reported similar PK profiles to that of α-MG. After i.v. administration, γ-MG exhibited a two-compartmental body model, indicating its distribution into the peripheral compartments. Both α-MG and γ-MG exhibited an intensive first-pass metabolism with rapid conjugation, fast distribution, and slow elimination after oral administration [108].

Intriguingly, the low bioavailability of α-MG and γ-MG was partly improved when they were administered as a form of an extract, despite still being low [108,109]. Therefore, we hypothesize that the PK parameters of α-MG and γ-MG may be influenced by other polyphenols present in the MPE. When administered as an extract, the total absorption of α-MG and γ-MG was not changed [108], or increased [109]; however, the conjugation was slower, resulting in increased exposure to free (unconjugated) compounds. Since beneficial biological activities of xanthones from MP were based on the free, unconjugated compounds, food supplements containing MPE would be preferred products over that containing pure xanthones [108].

Taken together, xanthones from MP, such as α-MG and γ-MG, have low oral absorption and are highly distributed into the peripheral compartments, but not in the brain. Additionally, these xanthones undergo phase II metabolism exhibiting a slow terminal elimination phase. The low bioavailability of the xanthones limits their pharmacological actions, especially in the brain. Interestingly, the usage of these xanthones as a form of an extract partially improves the PK parameters and bioavailability of the active components; however, these studies should be repeated in humans to fully elucidate the PK properties.

### 6.2. PK Profiles of MP-Derived Agents in Humans

Several reports have measured the PK profiles of extracts or xanthones from MP in humans. The bioavailability of xanthones from 100% mangosteen juice was determined in healthy adult participants [110]. A single ingestion of mangosteen juice with a high-fat breakfast led to absorption and partial conjugation of xanthones. Notably, both free and glucuronidated/sulfated xanthones of α-MG, γ-MG, 8-deoxygatanin, garcinones D and E, and gartanin were detected in the serum and urine following ingestion. It was also found that approximately 15.4% of total xanthones in the juice partitioned into mixed micelles during in vitro digestion. This observation was supported by a study using a human epithelial colorectal adenocarcinoma Caco-2 cell line. Transepithelial transport of α-MG and γ-MG was dependent on taurocholate micellarization in Caco-2 cells [111]. Collectively, these findings demonstrate that xanthones in mangosteen juice are absorbed when ingested with a high-fat meal, even though the release of xanthones from the pericarp particles may be limited during digestion [111].

Two randomized double-blind, placebo-controlled clinical trials with a similar design were performed to investigate the absorption of a mangosteen fruit-based functional beverage in healthy adults [112,113]. α-MG gradually increased to reach the maximum concentration after 1 h and sustained up to 6 h of the trial period [112,113]. These studies considerably contributed to our understanding of PK properties of mangosteen-based functional beverages in healthy adults; however, there were limitations in this study. The plasma samples were collected only up to 6 h after the ingestion of the product, and the xanthone metabolites were not investigated. In addition, the beverage included other components, such as aloe vera, green tea, and multivitamins, which might affect the effectiveness and bioavailability of α-MG [112].

In summary, these studies showed that xanthones in mangosteen juice are absorbed, especially when administered with a high-fat meal. Unfortunately, all the PK studies in humans have been conducted using mangosteen fruit-based products. Therefore, further studies using specific MPE or bioactive xanthones are necessary. Moreover, in order to assure therapeutic efficacy of MP-derived agents in AD, PD, and depression, solutions to improve their PK profiles with more distribution into the brain should be elucidated.

### 6.3. Proposed Solutions to Improve the PK Profiles of MP-Derived Agents

Among the bioactive xanthones, α-MG is the most studied compounds in the brain diseases; however, its efficacy may be limited due to the poor penetration through BBB [114]. In recent years, numerous studies have sought to overcome the poor bioavailability of α-MG in the brain and augment its potential clinical efficacy in the treatment of neurological diseases.

One study tested PK properties of the α-MG-loaded microemulsion in i.p. injected rodents. α-MG was universally distributed into almost all organs, including the liver, kidney, and brain. This study demonstrated that the microemulsion was an efficient delivery system for α-MG to cross BBB [115]. Another proposed delivery system was transferrin-modified liposomes (Tf(α-MG) liposome) administered through i.v. injection [114]. This liposome improved the bioavailability of α-MG in the plasma and augmented its delivery and accumulation in the brain. These results proved that the Tf(α-MG) liposome targeting the brain could be a potential carrier of α-MG for the treatment of AD [114]. However, i.p. and i.v. administrations may not be appropriate to apply to the actual treatment regimen of AD in humans [115]. Oral administrations of α-MG may possess limitations due to its hydrophobic nature, poor aqueous solubility and stability, low bioavailability, and lack of accumulation in the target organs including brain [116,117]. Therefore, more appropriate delivery system is absolutely required to improve the efficacy of orally administered α-MG. Noticeably, a soft capsule containing vegetable oil as a dispersion matrix of α-MG exhibited its quick absorbance after oral administration with a wide distribution in many tissues including brain [118].

Recently, advances in nanotechnology have promised an opportunity to overcome the obstacles in the treatment of various brain disorders including AD [119]. First, polyvinylpyrrolidone (PVP) was explored as a hydrophilic matrix to carry α-MG in the ethanol MPE (MPE:PVP nanofibers). These nanofibers were demonstrated to markedly increase the release rate and antioxidant activity of α-MG, improving the therapeutic effects offered by the ethanol MPE [16]. Furthermore, a nanoencapsulation of α-MG with a core of poly(ethylene glycol)-poly(l-lactide) nanoparticles [NP(α-MG)] improved the distribution of α-MG in the brain and liver, and specifically, enhanced the brain clearance of ^125^I-radiolabeled Aβ_1-42_ in a low-density lipoprotein receptor (LDLR)-dependent manner. LDLR mediates Aβ uptake and clearance by astrocytes; therefore, increased glial LDLR expression may promote Aβ degradation within the brain [120]. Consequently, the NP(α-MG) reduced Aβ deposition, attenuated neuroinflammatory responses, ameliorated neurologic changes, and reversed behavioral deficits in AD models [121]. Another biomimetic delivery system of α-MG is apolipoprotein E-reconstituted high-density lipoprotein nanocarrier (ANC-α-MG). It possessed improved brain delivery efficiency and high Aβ-binding affinity to intervene AD progression. As a result, ANC-α-MG decreased amyloid deposition, attenuated microgliosis, and rescued memory defects in mice, providing a promising platform for brain drug delivery for the treatment of AD [122].

Taken together, these studies highlight several solutions to improve the PK profiles of MP-derived agents for AD therapy, including nanotechnology to increase bioavailability and penetration through BBB. These novel techniques improving the delivery of MP-derived agents to the brain may also be applicable to drugs for a wide variety of other CNS disorders. However, it should be considered that enhanced bioavailability and wider distribution in tissues including liver may induce more toxicity. Safety profiles of MP-derived agents are discussed in the following section.

## 7. Safety Profiles of MP-Derived Agents

MP has been used as a traditional medicine for a long time; therefore, it is considered as generally safe, even in humans [13]. However, a comprehensive awareness of safety profiles is very important to further evaluate the therapeutic potential of MP-derived agents for disease treatments. In this section, we summarize the safety profiles of the agents in both animals and humans.

### 7.1. Safety Profiles of MP-Derived Agents in Animals

Cumulative studies have demonstrated that MP-derived agents show no acute or chronic toxicity. Single oral administration of MPEs fractionated with different percentages of ethanol up to 5000 mg/kg b.w. or α-MG up to 2000 mg/kg did not exhibit any signs or symptoms of toxicity, mortality, and behavioral changes in rodents during the study periods [25,123,124,125,126,127,128]. Similarly, daily oral doses up to 2000 mg/kg of the ethanol MPEs or Meratrim appeared to be safe and well-tolerated in rodents [124,125,126,129].

Nevertheless, the MPEs and α-MG may induce mortality in animals with increased dosages [109,123,129,130]. Several side effects have been reported, such as intestinal dysbiosis in several strains of mice [131,132], cardiac dysfunction, and disrupted erythropoiesis in zebrafish embryos [130]. The increased bioavailability of α-MG using techniques described above may enhance toxicity risks. In fact, side effects including significant b.w. loss, liver ascites, and high mortality were observed in mice after repeated i.p. administration of the α-MG-loaded microemulsion at 10 mg/kg for 10 days [115]. This may be due to the lipophilic nature and specific targeting property of α-MG, leading to its high accumulation in the liver and inducing hepatic injury [115]. Similarly, the enhanced distribution of α-MG in the liver was noticed in the other delivery systems of Tf-liposome and nanoencapsulation [114,121]. This may also increase the risk of α-MG-induced hepatotoxicity. Further evaluations of the safety profiles of these carrier systems are required before their clinical uses.

In light of the diverse findings, the ethanol MPEs and α-MG exhibited broad spectrum of safety profiles in animals. However, potential induction of adverse effects such as dysbiosis, cardiac dysfunction, and hepatic toxicity should be taken into account with increased dosages or repeated administrations.

### 7.2. Safety Profiles of MP-Derived Agents in Humans

Although toxicity profiles of MP-derived agents are generally safe and tolerable in most animal studies, it is difficult to extrapolate their safety profiles to humans. Unfortunately, there are currently no clinical studies for these agents in patients with AD, PD, or depression; therefore, their safety profiles in humans lack evidence. Nevertheless, these products have been studied in individuals with obesity because of their effects on weight management [101]. In an 8-week randomized, double-blind, placebo-controlled study of a juice blend containing mangosteen whole fruit puree, no side effects or significant laboratory or electrocardiogram changes were reported in 44 obese individuals [133]. Similarly, two randomized, double-blind, placebo-controlled clinical trials evaluated safety profile of a product similar to Meratrim in 100 overweight participants, and reported no alterations across organ function panels and multiple vital signs, or major adverse events [134,135]. Based on these findings, mangosteen-based products may be considered tolerable in obese individuals.

In addition, several studies have indicated the safety of mangosteen-based products in healthy individuals. A randomized, double-blind, placebo-controlled clinical trial enrolling 30 men and 30 women determined no side effects on either immune, hepatic, or renal functions following long-term consumption of a mangosteen fruit-based beverage [136]. In another study, the safety profile of orally administered polar fraction from ethanol MPE was investigated in 11 healthy Thai volunteers. Only minor and tolerable adverse effects were documented during the 24-week period without serious side effects, liver damage, or kidney dysfunction in the participants. However, this study had limitations because it did not include a placebo control group, had a small sample size, and administered a relatively small dose of the MPE; therefore, it was difficult to validate the safety profile of MPE administration [24].

In a RCT conducted using encapsulated MP powder in schizophrenia patients, MP was well tolerated in the participants with high adherence during the study period. Nonetheless, the relatively small number of patients and nature of the population may lead to a possible underestimation of adverse events [102]. Remarkably, a case of severe lactic acidosis in the individual using mangosteen juice as a dietary supplement was reported. However, no cause-effect relationship was determined, mainly because the mangosteen was not a single constituent administered in this case [137].

Collectively, these findings indicate positive safety profiles of mangosteen-based products in humans. However, most studies have investigated the safety of mangosteen fruit-based beverages, not MP-derived products directly. Moreover, most studies employed healthy adults or patients with obesity; no studies have studied in patients with AD, PD, or depression. Therefore, further studies should be conducted to assess overall safety profiles of MP-derived agents in clinical conditions. Since the safety of agents containing MPE or powder and their xanthones is not fully understood yet, the wide consumption of these products should be reconsidered to avoid consumers misled by their overall safety and efficacy [138].

## 8. Conclusions

**Figure 2:**
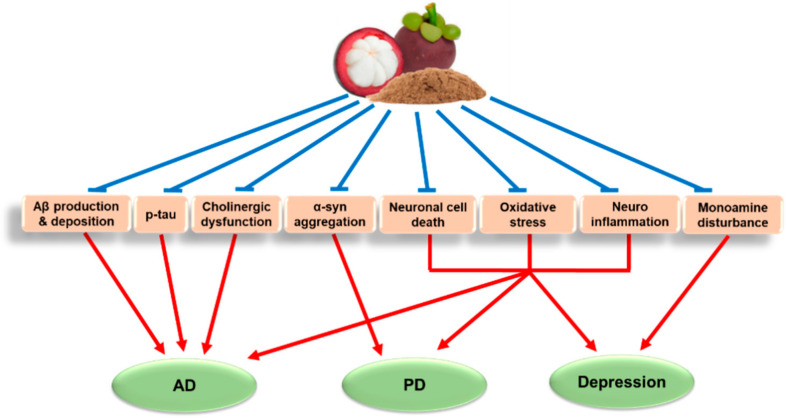
Mechanisms of actions of mangosteen pericarp (MP)-derived agents to combat Alzheimer’s disease (AD), Parkinson’s disease (PD), and depression. MP-derived agents, including dietary supplements, extracts, and bioactive xanthones isolated from MP, inhibit Aβ production and deposition, tau hyperphosphorylation (p-tau) and cholinergic dysfunction in various in vitro and in vivo AD models. These agents abate α-synuclein (α-syn) aggregation in PD models, and diminish monoamine disturbances in depression models. Moreover, the agents mitigate neuronal cell death, oxidative stress, and neuroinflammation, which further contribute to their pharmacological effects to combat progression of these diseases.

Although MP has been used as a traditional medicine for hundreds of years in Southeast Asia for a variety of medical conditions, the exact pharmacological functions of MP and their underlying action mechanisms in neurodegenerative and mental diseases such as AD, PD, and depression remain to be elucidated. There have been considerable advances in our efforts to dig deeper in the mechanisms of actions of MP-derived agents in fighting the brain disorders. In this review, we discussed in vitro and in vivo pharmacological effects of MP-derived agents in various AD, PD, and depression models along with their pharmacokinetic and safety profiles. Accumulated preclinical studies demonstrated significant roles of MP and its xanthone derivatives, especially α-MG and γ-MG, in preventing progression of these diseases. Intriguingly, the multiple pharmacological effects of these agents are commonly associated with neuroprotective, antioxidant, and anti-inflammatory activities. Additionally, these agents target Aβ production and deposition, and cholinergic dysfunction in AD; α-syn aggregation in PD; and monoamine dysregulation in depression. The overall mechanisms of actions of MP-derived agents in these diseases are summarized in Figure 2. Even though the preclinical evidence is promising to exhibit pharmacological effects of MP-derived agents in AD, PD and depression models, there is only limited information of mental health outcomes available in humans. Clinical data will be absolutely required to validate their therapeutic efficacy in patients with these diseases.

α-MG and γ-MG show orally low bioavailability and poor BBB penetration, which may reduce the efficacies of these compounds in the brain disorders. Interestingly, administration as a form of MPE or using novel delivery systems including nanotechnology has improved PK parameters of the single bioactive compound. Ethanol MPE and α-MG are considered to be safe and well-tolerated in animals. In addition, mangosteen-based products exhibit good safety data in humans. However, further studies with various MPEs and bioactive xanthones should be performed to confirm their safety in patients with AD, PD, or depression. In addition to α-MG and γ-MG, other xanthones with potential therapeutic benefits should be further investigated to decipher their efficacy, pharmacokinetic and safety profiles in the brain disorders.

## Figures and Tables

**Table 1:** Pharmacological effects of MP-derived agents in Alzheimer’s disease models.

No.	Agents	Experimental Models	Experimental Conditions	Results	References
1	Water-soluble partition of methanol MPE	SK-N-SH cells	Aβ_1-42_	↓ Neurotoxicity ↓ Caspase-3 ↓ ROS	[73]
2	Butanol fraction of methanol MPE	Primary cultured rat cortical neurons	NMDA Aβ_25-35_	↓ Neurotoxicity & apoptotic events ↓ ROS	[74]
		Rat brain homogenates	Fe^2+^/ascorbic acid	↓ Lipid peroxidation	
		Cell-free bioassay		↓ β-Secretase activity	
3	Water MPE	Primary cultured rat cortical neurons	NMDA Aβ_25-35_	↓ Neurotoxicity & apoptotic events ↓ ROS	[75]
		Rat brain homogenates	Fe^2+^/ascorbic acid	↓ Lipid peroxidation	
		Cell-free bioassay		↓ β-Secretase activity ↓ AChE activity	
		ICR mice	Scopolamine	↓ Memory impairment	
4	Water/50% ethanol MPE	NG108-15 cells	H_2_O_2_	↓Oxidative neurotoxicity Free radical scavenging activity	[76]
5	Water-soluble partition of ethanol MPE	SK-N-SH cells	H_2_O_2_ PCBs	↓Oxidative neurotoxicity ↓ Caspase-3 ↓ ROS ↓ AChE activity	[77]
		ICR mice	Scopolamine	↓ Memory impairment ↓ Brain ROS ↓ Caspase-3	
6	MP diet	3×Tg-AD mice	NA	↓ Aβ deposition ↓ Phosphorylated tau ↓ Memory impairment	[78]
		B6 mice		↓ Systemic IL-6 ↑ BDNF level ↓ Phosphorylated tau ↓ Cognitive impairment	
	MP	OHSC		↓ Neurotoxicity ↑ BDNF level	
7	50% Ethanol MPE	Male SA mice	Streptozotocin	↑ Antioxidant parameters: superoxide dismutase, glutathione peroxidase, glutathione, and catalase ↓ AChE levels ↓ Cognitive & memory impairment	[79]
8	α-MG, γ-MG, gartanin, garcinone C	HT22 cells	Glutamate	↓ Neurotoxicity ↑ HO-1 level ↓ DPPH radicals	[80]
		*E. coli*/Cell-free bioassay		↓ Self-induced Aβ aggregation	
		Cell-free bioassay		↓ β-Secretase activity	
9	α-MG	Primary cultured rat cortical neurons	NA	↓ β- & γ-Secretase activity ↓ Aβ_1-40_ & Aβ_1-42_ production	[81]
10	α-MG	Primary cultured rat cortical neurons	Aβ_1-40_ or Aβ_1-42_ oligomers	↓ Neurotoxicity ↓ Aβ fibril formation & pre-formed fibrils	[82]
11	*γ*-MG	Primary cultured rat cortical neurons	H_2_O_2_ or xanthine/xanthine oxidase	↓ Oxidative neurotoxicity ↓ ROS ↓ DNA fragmentation ↓ Caspases-3 & 9	[83]
		Rat brain homogenates	Fe^2+^/ascorbic acid	↓ Lipid peroxidation & DPPH radicals	
		Cell-free bioassay		↓ β-Secretase activity	
		ICR mice	Scopolamine	↓ Memory impairment	
12	α-MG, γ-MG, mangostanol, 3-isomangostin, & garcinone C	Cell-free bioassay	NA	↓ AChE activity	[84]
13	α-MG	Female B6 mice	LPS	↓ IL-6, COX-2 & TSPO	[20]

Aβ, amyloid beta; AChE, acetylcholinesterase; α-MG, α-mangostin; B6, C57BL/6J; BDNF, brain-derived neurotrophic factor; COX-2, cyclooxygenase-2; DPPH, 2,2-diphenyl-1-picrylhydrazyl; E. coli, Escherichia coli; γ-MG, γ-mangostin; GSH, glutathione; HO-1, heme oxygenase-1; ICR, Institute for Cancer Research; IL, interleukin; LPS, lipopolysaccharide; MP, mangosteen pericarp; MPE, mangosteen pericarp extract; NA, not applicable; NMDA, N-methyl-D-aspartate; OHSC, organotypic hippocampal slice culture; PCBs, polychlorinated biphenyls; ROS, reactive oxygen species; SA, Swiss albino; TSPO, 18 kDa translocator protein.

**Table 2:** Pharmacological effects of α-mangostin and γ-mangostin in Parkinson’s disease models.

No.	Agents	Experimental Models	Experimental Conditions	Results	References
1	α-MG	SH-SY5Y cells	MPP^+^	↓ Apoptosis ↓ ROS	[86]
2	α-MG	SH-SY5Y cells	Rotenone	↓ Cell death ↓ Caspases-3 & 8 ↓ ROS ↓ Mitochondrial dysfunction ↓ Aggregation of α-syn and TH loss	[87]
3	α-MG	Primary rat microglia cells	α-Syn	↓ ROS ↓ TNF-α, IL-1β, IL-6, NO, NF-κB & NADPH oxidase	[88]
		Primary rat mesencephalic neuron-glia co-culture	α-Syn	↓ DAergic neuronal cell death	
4	α-MG	Female B6 mice	LPS	↓ IL-6, COX-2 & TSPO	[20]
5	α-MG	Adult male SD rats	Rotenone	↓ MDA, nitrite ↑ GSH ↓ Phosphorylated α-syn ↓ TH^+^-DAergic neuronal loss in SNpc ↑ Locomotor activity ↑ Neuromuscular function	[89]
6	γ-MG	SH-SY5Y cells	6-OHDA	↓ Neurotoxicity ↓ Apoptosis ↑ Antioxidant potential	[90]

6-OHDA, 6-hydroxydopamine; α-MG, α-mangostin; α-syn, α-synuclein; DAergic, dopaminergic; γ-MG, γ-mangostin; GSH, glutathione; IL, interleukin; MDA, malondialdehyde; MPP^+^, 1-methyl-4-phenylpyridinium; NF-κB, nuclear factor kappa B; NO, nitric oxide; ROS, reactive oxygen species; SD, Sprague Dawley; SNpc, substantia nigra pars compacta; TH, tyrosine hydroxylase; TNF-α, tumor necrosis factor-α.

**Table 3:** Pharmacological effects of MP-derived agents in depression models.

Agents	Experimental Models	Experimental Conditions	Results	References
Ethyl acetate MPE	FSL rats	Acute treatment Chronic treatment	Antidepressant-like effect Antidepressant & pro-cognitive effects Prominent serotonergic action ↓ Hippocampal lipid peroxidation	[92]
Ethyl acetate MPE/α-MG	Pregnant female SD rats	MIA	Antidepressant-like effect in schizophrenia	[98]
Mangosteen-based products	Healthy adults	RCT	Positive impact on POMS scores	[99]
Mangosteen-based products (Meratrim)	Overweight subjects	RCT	↓ Body weight ↓ Total mood disturbance	[101]
Encapsulated MP powder	Patients with schizophrenia or schizoaffective disorder	RCT	↓ PANSS ↓ MADRS	[102]
Water MPE	Patients with bipolar depression	RCT	No published results	[103]
α-MG	Mice	TST	Antidepressant-like activity (reversed by pretreatment with HAL, bicuculline & p-CPA) ↑ Brain DA, 5-HT & GABA levels	[97]
α-MG	Female B6 mice	LPS	↓ IL-6, COX-2 & TSPO	[20]

α-MG, α-mangostin; DA, dopamine; FSL, Flinders Sensitive Line; GABA, gamma-aminobutyric acid; HAL, haloperidol; MIA, maternal immune-activation; MADRS, Montgomery Åsberg Depression Rating Scale; MP, mangosteen pericarp; MPE, mangosteen pericarp extract; PANSS, Positive and Negative Syndrome Scale; POMS, Profile of Mood States; p-CPA, *p*-chlorophenylalanine; RCT, randomized controlled trial; SD, Sprague Dawley; TST, tail suspension test.
